# Effect of Acid Hydrolysis Conditions on the Extraction of Cellulose Nanocrystals

**DOI:** 10.3390/polym17101313

**Published:** 2025-05-12

**Authors:** Víctor M. Serrano-Martínez, Henoc Pérez-Aguilar, María Pilar Carbonell-Blasco, Avelina García-García, Francisca Arán-Ais, Elena Orgilés-Calpena

**Affiliations:** 1 Footwear Technology Centre, Campo Alto Campground, 03600 Alicante, Spain; 2 MCMA Group, Department of Inorganic Chemistry and Institute of Materials, University of Alicante, Carretera de Sant Vicent del Raspeig, s/n, 03609 Sant Vicent del Raspeig, Spain

**Keywords:** cellulose extraction, nanocrystals, lignocellulosic biomass, mechanical strength, acid hydrolysis, rice straw waste, renewable resource

## Abstract

The quest for sustainable and efficient methods for extracting cellulose nanocrystals (CNCs) remains critical in the development of advanced materials. This study refines the acid hydrolysis process for cellulose derived from rice straw, an abundant agricultural waste, focusing on the relationship between hydrolysis parameters and CNC properties. The research identifies conditions that enhance crystallinity and colloidal stability while effectively transforming amorphous cellulose into nanocrystals. Advanced characterization techniques, including XRD, SEM, TEM, and zeta potential measurements, provided insights into the structural and colloidal properties of CNCs. The findings demonstrate the influence of reaction temperature, time, and acid concentration on CNC quality, with optimized conditions yielding nanocrystals with enhanced stability and well-defined morphology. This study underscores the potential of rice straw as a renewable resource, advancing the circular economy by promoting the use of sustainable materials in high-value applications such as composite reinforcement and coatings.

## 1. Introduction

Cellulose stands as the most abundant biopolymer on Earth, integral to the development of renewable and sustainable materials due to its biodegradability and low environmental and human health risks. Its transformation into nanocellulose, including nanofibers and nanocrystals, has expanded its application from traditional uses in textiles and papermaking to advanced materials engineering fields [1]. This study focuses on cellulose nanocrystals (CNCs), which, like cellulose nanofibers (CNFs), possess unique properties such as high mechanical strength, chemical stability, and abundant surface functionality, which make them ideal for a wide range of applications, including sustainable water purification, energy storage, and high-performance composites [2]. In the medical field, it has shown potential in drug delivery systems, wound healing materials, and scaffolds for tissue engineering due to their biocompatibility, high surface area, and mechanical strength [3]. Its versatility is further enhanced by various preparation methods, including both top-down and bottom-up approaches, allowing for the control of fibre dimensions and properties to meet specific application needs. Top-down methods, such as acid hydrolysis and mechanical disintegration, are commonly used to produce CNCs by breaking down larger cellulose structures into nanoscale crystalline domains. In contrast, bottom-up approaches involve the assembly of nanocellulose structures from molecular precursors, though these are less commonly used for CNCs [4]. The production of nanocellulose from different biomass sources and through various mechanical processing methods has been explored to optimise its properties and sustainability [5]. These efforts underscore the importance of cellulose as a renewable resource in the transition towards green and sustainable energy technologies, where nanocellulose-based materials are being developed for use as scaffolds, sorbent materials, adhesives, and suspending agents [6]. Furthermore, the combination of nanocellulose with other nanomaterials, such as graphene derivatives, has led to the creation of hybrids with enhanced physicochemical properties, opening new avenues for their application in electronics, thermal management, and electromagnetic shielding [7,8]. The ongoing research and development in the field of nanocellulose underscore its significance as a renewable resource and its transformative potential in materials engineering and revaluation [9].

The extraction and preparation of CNFs and CNCs have evolved significantly, employing various methods to harness their potential in industrial applications. Traditional mechanical methods, such as high-pressure homogenisation, have been utilised to isolate cellulose micro-/nanofibres directly in non-aqueous media like propylene glycol, maintaining high crystallinity and thermal stability, crucial for developing nanocomposites with hydrophilic matrices [10]. Similarly, acid hydrolysis, a conventional technique, has been refined to extract CNCs with high purity and thermal stability from microcrystalline cellulose, using a two-stage process involving ionic liquid pretreatment and solid acid hydrolysis, demonstrating the method’s effectiveness in preserving the rodlike morphology and crystallinity of CNCs [11]. Innovative approaches have also been explored for extracting high-purity cellulose from agri-food waste, employing a three-step chemical procedure followed by acid hydrolysis to obtain cellulose nanocrystals, showcasing the method’s versatility across different waste feedstocks [12]. Electrospinning has also emerged as a promising technique for producing cellulosic nanofibers, overcoming the limitations of cellulose’s poor solubility through the use of various solvents, thus enabling the fabrication of nanofibers with enhanced surface area and mechanical properties for applications in tissue engineering and drug delivery [13]. Recent advancements include the use of supercritical CO_2_ (ScCO_2_) for isolating CNFs from carpet wastes, offering a green approach that enhances yield and quality [14], and the TEMPO-mediated oxidation method, which has proven effective in pre-treating cellulose from Ficus natalensis barkcloth for CNF production, achieving significant morphological changes and higher crystallinity [3]. These current methods, alongside developments in spinning techniques for recycling cellulose waste into valuable new materials [15] and the exploration of energy-efficient approaches for CNF production [16], highlight the ongoing innovation in cellulose nanofiber extraction techniques, catering to the demand for sustainable and high-performance materials in various industries.

Acid hydrolysis per se is considered superior to conventional methods for the extraction of CNFs and CNCs due to several key advantages. Firstly, acid hydrolysis, particularly using sulphuric acid, is favoured for its ability to produce CNCs with high crystallinity and stability in a relatively short time. This method typically results in needle-like structures with controlled morphology, which is crucial for applications requiring specific dimensional and thermal stability characteristics [17]. The process involves treating cellulose sources with sulphuric acid under controlled conditions, leading to the hydrolysis of amorphous regions while preserving the crystalline regions, thereby producing CNCs with desirable properties [18]. In comparison to other methods, such as mechanical or enzymatic hydrolysis, acid hydrolysis offers a faster route to obtaining nanocellulose with higher yield and crystallinity [19]. For instance, enzymatic hydrolysis, while being less aggressive towards the environment, tends to be slower and may not always provide the high crystallinity and thermal stability that acid hydrolysis can. Moreover, mechanical methods, which include high-intensity ultrasonication or steam explosion processes, may require more energy and still not match the efficiency of acid hydrolysis in terms of yield and quality of the nanocellulose produced [20]. Other scientists have explored alternative methods, such as using ionic liquids or ScCO_2_ treatment, which present environmentally friendly options but may involve more complex processes or equipment. For example, the use of ionic liquids has been reported to produce CNCs with similar physical properties to those prepared under conventional conditions, offering a mild alternative to acid hydrolysis [17]. Similarly, ScCO_2_ treatment has been shown to enhance the isolation yield of CNFs from carpet wastes, producing nanocellulose with higher thermal stability and a cleaner chemical composition [21]. However, these methods still face challenges in scalability and cost-effectiveness compared to acid hydrolysis. In summary, acid hydrolysis remains a preferred method for extracting cellulose nanofibers due to its efficiency, ability to produce nanocellulose with high crystallinity and thermal stability, and the controlled morphology of the resulting CNCs. While alternative methods offer environmental benefits and novel properties, acid hydrolysis provides a balance of efficiency, control, and applicability across various applications [14,22].

Building upon a previously established process for the extraction of cellulose from rice straw using a steam explosion technique [23], the current research seeks to advance this foundational work. The focus is on the precise conversion of cellulose into CNCs through acid hydrolysis, exploring the effects of varying time, concentration, and temperature on the quality of CNCs. A Central Composite Design (CCD) statistical analysis has been used to create an experimental framework to determine the optimal hydrolysis conditions. The characterisation of the physicochemical properties of CNCs is conducted using a comprehensive suite of analytical techniques. X-ray Diffraction (XRD) insights into the crystalline structure are paired with morphological details revealed by Scanning Electron Microscopy (SEM) and Transmission Electron Microscopy (TEM), while zeta potential measurements indicate surface charge properties and dispersibility.

The outcome of this work will yield a detailed understanding of CNCs derived from rice straw, establishing a benchmark for the production of nanocellulose and thus contributing to the advancement of materials science. Thus, this investigation focuses on exploring CNCs to meet the specific requirements of the footwear industry. The primary goal is to develop innovative materials that enhance the mechanical properties of footwear, offering more robust and durable products. By tailoring these advancements to the needs of footwear manufacturing, the study not only aims to push the boundaries of material science but also to optimise the use of agricultural by-products, thereby minimising waste and enhancing resource efficiency within the sector. At the same time, it is important to note that beyond the footwear sector, CNCs hold great potential in diverse areas such as biomedicine, flexible electronics, packaging, water treatment, and as reinforcing agents in biocomposites, due to their versatility and tunable surface chemistry [24]. These diverse applications highlight the transformative potential of CNCs in advancing sustainable product development across various industries.

## 2. Materials and Methods

### 2.1. Materials

**Figure 1:**
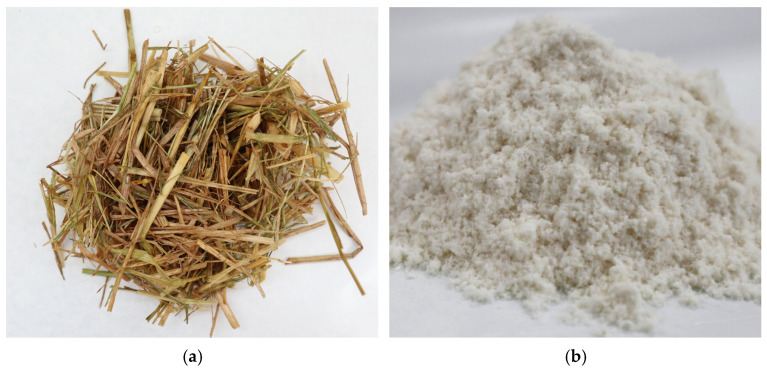
(**a**) Chopped rice straw before processing it and (**b**) extracted cellulose used as a raw material.

Cellulose, extracted from rice straw provided by Unió Llauradors i Ramaders (Valencia, Spain), was used as the primary material for the production of cellulose nanocrystals (CNCs) via acid hydrolysis. The rice straw, sourced from the Albufera of Valencia, a significant rice-growing area known for sustainable agriculture practices, was initially cut into pieces of approximately 10 mm to maximize the surface area exposed during the subsequent pre-treatments. These pre-treatments, outlined in the previous investigation [23], included a 30-min steam explosion, 3 h of oxidation, and 1 h of bleaching, preparing the cellulose for the subsequent conversion process. To better illustrate their physical characteristics, Figure 1 shows both rice straw before processing and extracted cellulose.

For the acid hydrolysis step, 95% sulphuric acid obtained from Sigma-Aldrich (Darmstadt, Germany) was employed. This reagent was diluted to a 50% concentration, sufficient to cover all experimental needs. All solutions and mixtures were prepared using distilled Milli-Q water to maintain the consistency and reliability of the experimental outcomes. The detailed methodology for the acid hydrolysis process is discussed in the following section.

### 2.2. Extraction of Cellulose Nanocrystals from Rice-Straw-Extracted Cellulose

The preparation of cellulose nanocrystals (CNCs) from rice straw extracted cellulose builds upon previous work that extensively optimised the extraction of cellulose from rice straw using a steam explosion technique [23]. This prior research established the foundational material for the current study, demonstrating how steam explosion effectively disrupts the lignocellulosic matrix to release cellulose. This process provides a purified cellulose source with the potential for further transformation into nanocrystals through acid hydrolysis.

The production of CNCs was initially structured around a Central Composite Design (CCD). This statistical approach was selected to systematically explore the response surface of key experimental variables [25]—reaction temperature, time, and sulphuric acid concentration—and to optimise the hydrolysis process. CCD offers a powerful framework for simultaneously testing multiple variables while minimising the number of experimental runs. This methodology aimed to selectively remove amorphous cellulose regions while preserving crystalline domains, yielding CNCs with desirable structural and physicochemical properties.

Based on prior research [26,27,28,29,30,31], the selected working ranges for the variables were as follows. Temperatures were set to range between 39 °C and 81 °C, allowing for the evaluation of mild to high-temperature conditions relevant to the hydrolysis process. Reaction times were varied from 32 to 88 min to capture the effects of both shorter and prolonged hydrolysis durations. Sulphuric acid concentrations were tested between 25 and 85.36 mL of 50% H_2_SO_4_ per gram of cellulose, representing a broad range of acid strengths to determine the balance between efficient hydrolysis and potential cellulose degradation. Table 1 summarises the experimental conditions generated by the CCD.

**Figure 2:**
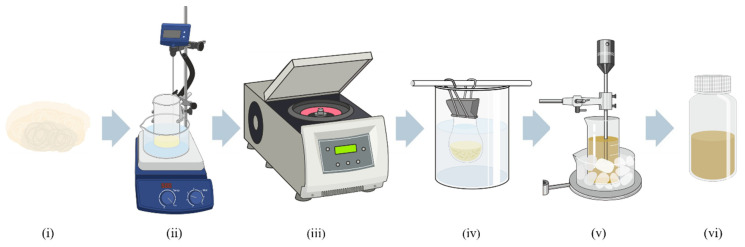
Schematic representation of the acid hydrolysis process for the extraction of cellulose nanocrystals: (**i**) cellulose sample, (**ii**) acid hydrolysis reaction under stirring and controlled temperature, (**iii**) centrifugation, (**iv**) dialysis, (**v**) ultrasonication, and (**vi**) final CNC suspension.

For the hydrolysis reaction, a sufficient quantity of 50% sulphuric acid (H_2_SO_4_) was pre-prepared to ensure consistency throughout the experimental runs, thereby minimising potential preparation errors during repeated setups. For each experiment, approximately 1 g of the processed cellulose was subjected to acid hydrolysis under controlled conditions. The sulphuric acid-to-cellulose ratio was adjusted for each sample based on the experimental design, optimised through a response surface methodology, with ratios ranging between 1:14.65 and 1:85.36. This optimisation sought to balance efficient hydrolysis with minimal degradation, facilitating the transformation of cellulose fibres into nanocrystals with enhanced properties. Specifically, the process effectively removes impurities and amorphous cellulose, yielding a purer nanofiber product with improved crystallinity and stability, as demonstrated in similar studies [20]. The entire hydrolysis process is schematically illustrated in Figure 2 for clarity.

Initially, the acid-to-cellulose mixture was prepared in a 1 L beaker, heated, and stirred at 600 rpm (ii). Reaction temperatures ranged from 39 °C to 81 °C, with durations of 32 to 88 min, as dictated by the CCD framework, to facilitate the hydrolysis process. Following the reaction’s completion, the mixture was immediately diluted with distilled water in a 1:10 ratio (e.g., 50 mL H_2_SO_4_ diluted with 500 mL H_2_O) to quench the hydrolysis reaction and cool the mixture. This step was critical to halt the acid action and stabilise the mixture for downstream processing.

The diluted hydrolysis mixture was centrifuged at 6000 rpm for 10 min to separate the solid cellulose nanocrystals from the liquid phase (iii). This centrifugation step was repeated until the pH of the supernatant stabilised near neutrality. Subsequently, the nanocrystals were purified via dialysis (iv) against distilled water using cellulose membrane tubing (76 mm width). The dialysis lasted five days, with water changes performed twice daily, until the medium’s pH stabilised between 6.5 and 7. To ensure proper dispersion for further analysis, the CNC suspensions underwent ultrasonication using a Branson Digital Sonifier model 102C (v) (Branson Ultrasonics Corporation, Danbury, CT, USA). The ultrasonication process lasted 10 min at 50% amplitude with intermittent pulses (10 s on, 10 s off), resulting in a total duration of 20 min. This step was performed in a cold-water bath to prevent overheating, ensuring the CNCs were evenly dispersed without structural damage.

While the CCD provided a strong foundation for exploring a wide range of experimental conditions, certain challenges inherent to the hydrolysis process influenced the final scope of the study. Variability in results, particularly under extreme conditions within the design space, led to inconsistencies that affected the reproducibility of data across some samples. These observations underscored the sensitivity of acid hydrolysis to subtle changes in parameters such as temperature and acid concentration, which can significantly impact CNC quality.

To ensure a comprehensive understanding of the effects of acid hydrolysis conditions, five samples—PE3, PE5, PE7, PE9, and PE18—were selected for detailed characterisation. These samples were chosen as they represent key variations in hydrolysis parameters, encompassing a range of temperatures, acid concentrations, and reaction times, while consistently demonstrating properties indicative of high-quality CNCs. Their selection was guided by their balanced performance in terms of crystallinity, colloidal stability, and structural characteristics, providing a representative dataset for exploring the interplay between processing conditions and CNC properties.

By narrowing the scope to these representative samples, the study retains its scientific integrity and provides valuable insights into CNC production. This approach ensures that the findings are robust, reproducible, and relevant for advancing the understanding of acid hydrolysis as a method for CNC extraction.

Zeta potential measurements and SEM and TEM analyses were carried out on CNC suspensions in dispersion to avoid aggregation, which commonly occurs during drying processes. Thus, ensuring that the structural and morphological properties of the CNCs were accurately characterised.

### 2.3. Experimental Techniques

The characterisation of the cellulose fibres and lignin from the waste solutions generated during the different steps has been carried out using the following:

#### 2.3.1. Scanning Electron Microscopy (SEM)

The morphological analysis of CNCs was conducted using a high-resolution scanning electron microscope, model JEOL IT500HR/LA (JEOL Ltd., Tokyo, Japan), equipped with an energy-dispersive X-ray spectroscopy (EDS) detector. This instrument features a field emission gun, achieving resolutions of 1.5 nm at 30 kV and 4.0 nm at 1 kV, and operates within an acceleration voltage range of 0.5 to 30 kV. The samples were prepared by depositing a drop of the CNC suspension onto a conductive substrate, followed by drying under ambient conditions. Prior to imaging, the samples were coated with a thin layer of gold to enhance conductivity and image quality. The SEM analysis provided detailed insights into the surface morphology and size distribution of the CNCs [32].

#### 2.3.2. Transmission Electron Microscopy (TEM)

For a more in-depth examination of the internal structure and size of the CNCs, TEM analysis was performed using a JEOL JEM-1400 Plus (JEOL Ltd., Tokyo, Japan) transmission electron microscope operating at 120 kV. This instrument is equipped with a Gatan Orius SC600 camera (Gatan Inc., Pleasanton, CA, USA) for high-resolution image acquisition, achieving a point resolution of 0.38 nm and a line resolution of 0.2 nm. Sample preparation involved placing a drop of the CNC suspension onto a carbon-coated copper grid, followed by drying at room temperature. The TEM images facilitated the observation of the nanocrystals’ shape, size, and dispersion state, providing complementary information to the SEM analysis [33].

#### 2.3.3. X-Ray Diffraction (XRD)

The A non-destructive approach was adopted for the quantitative assessment of nanocrystals. This involved using wide-angle X-ray diffraction (XRD) technology, specifically a Bruker D8-Advance Göebel mirror system (Bruker AXS GmbH, Karlsruhe, Germany) designed for non-flat samples. The system, which includes a high-temperature chamber capable of reaching 900 °C, was coupled with a Siemens Bruker Kirstalloflex K 760-80F X-ray generator (Bruker AXS GmbH, Karlsruhe, Germany). This generator operates at a power of 3000 W, with an adjustable voltage ranging from 20–60 KV and a current between 5–80 mA and is equipped with a copper anode XR tube [34]. The crystallinity of the samples was determined using the crystallinity index (*CI*), calculated as per the methodology described by Khan et al. [35], which is defined as:

(1) $$CI=\frac{A_{c}}{A_{c}+A_{a}},$$

where *Ac* is the area under crystalline peaks, and Aa is the area of amorphous hollows. This approach involved extracting individual crystalline peaks through a curve-fitting process from diffraction intensity profiles using a tool for fitting peaks included in OriginPro 2021 software (OriginLab Corporation, Northampton, MA, USA), with Gaussian functions assumed for each peak. Iterations were repeated until a maximum F number, indicative of a high correlation coefficient (R^2^ value of 0.997), was achieved. Furthermore, the crystallite size was determined using the Scherrer Equation [36], given by:

(2) $$D_{p}=\frac{0.94\lambda }{\beta \cdot cos\theta },$$

where *Dp* is the average crystallite size, β is the line broadening in radians (FWMH), *θ* is the Bragg angle, and *λ* is the X-ray wavelength. This formula is widely recognised in X-ray diffraction analysis for estimating the sizes of crystallites in a sample.

Finally, diffractograms were indexed using peak positions corresponding to cellulose Iβ (ICDD patent PDF-2 database, File no 00-050-2241) [37,38]. Additional details regarding the analysis and interpretation of XRD data can be found in the Supplementary Material.

#### 2.3.4. Zeta Potential

For the measurement of zeta potential, the Zetasizer Nano ZS from Malvern Panalytical (Malvern Panalytical Ltd., Malvern, UK)., equipped with Zetasizer Software version 7.11, was employed. The analysed cellulose nanocrystal samples, after being neutralised and sonicated, were diluted to a concentration of 0.1% *v*/*v*. Prepared in a DTS1070 cell and using purified water as the dispersant, samples were maintained at a constant temperature of 25 °C. Each sample underwent a 2-min equilibration period before analysis. The process involved 10 to 100 iterations to ensure consistent zeta potential readings, with automatic adjustment of attenuation. The Smoluchowski function F(ka) was selected for results interpretation, appropriate for systems with low electrical conductivity, ensuring precision and reproducibility in the measurement of colloidal stability [31].

This technique is essential for evaluating the stability of cellulose nanocrystal suspensions, as the zeta potential provides insight into the surface charge of the particles and their tendency to aggregate or repel in solution. Accurate zeta potential measurements are crucial for assessing the colloidal stability of nanofibres, influencing the dispersion and behaviour of the crystals in various applications.

#### 2.3.5. Statistical Analysis

The statistical analysis for the Central Composite Design (CCD) was conducted using STATGRAPHICS Centurion v18 software, with a confidence level of 95% (*p* < 0.05) [39]. Although a complete statistical analysis covering all experimental conditions was not performed, the CCD framework provided a structured methodology to define the hydrolysis parameters and guide the selection of representative samples. This type of analysis is essential for understanding the influence of various experimental factors on the dependent variables, such as crystallinity, cellulose content, and the yield of the extracted cellulose nanocrystals.

The CCD is an advanced statistical technique used to model and optimise processes with multiple variables. In this study, it was employed to systematically explore the response surface of experimental variables, enabling the identification of key conditions that influenced CNC properties. The factors included reaction times, acid concentrations, and reaction temperatures, which were evaluated across a range of values to define a broad experimental space [40]. While not all CCD-generated conditions were fully analysed, this approach allowed the study to focus on a subset of samples representing diverse conditions within the design space.

Multifactor and simple ANOVA are commonly used in conjunction with CCD to study the influence of selected factors on dependent variables. The ANOVA methodology breaks down the total variability of the system into components attributable to each factor and their interactions, providing a clear understanding of how these variables influence outcomes. Key metrics in ANOVA include the F-ratio and *p*-value, which compare the variability between treatment groups with the variability within groups. A *p*-value lower than 0.05 indicates that the observed effects are statistically significant and not due to chance. Although these metrics are fundamental to CCD analysis, they were not applied in this study due to the focused nature of the selected dataset [41].

In summary, the use of a Central Composite Design in this study provided a robust framework for exploring the effects of acid hydrolysis parameters. By narrowing the focus to representative conditions, the CCD methodology enabled the identification of key trends and the generation of insights applicable to CNC optimisation. This structured approach lays the foundation for further refinement of hydrolysis processes and their potential scalability to industrial levels [42].

## 3. Results and Discussion

The impact of acid hydrolysis conditions on the stability and structural properties of cellulose nanocrystals (CNCs) derived from rice straw was thoroughly investigated. The selected samples—PE3, PE5, PE7, PE9, and PE18—were subjected to a detailed characterisation to elucidate the relationship between colloidal stability, crystallinity, and internal structure. This comprehensive analysis enables the establishment of a solid framework for optimising CNC production and tailoring their properties for specific applications.

Zeta potential measurements were performed on all CNC suspensions. These measurements are critical for understanding the electrostatic interactions governing particle dispersion in suspension. Zeta potential values greater than −30 mV (absolute value) are considered indicative of sufficient repulsion forces to prevent agglomeration, thereby ensuring long-term stability [43,44]. The results, presented in Table 2, reveal significant variations across the selected samples, reflecting the influence of hydrolysis conditions on their surface charge and stability.

The zeta potential for PE3, prepared under relatively mild conditions (60 °C, 60 min, 50 mL H_2_SO_4_), exhibited a zeta potential of −29.0 mV, indicating moderate colloidal stability. This value, while close to the threshold of stability (−30 mV), suggests a slight limitation in the removal of amorphous cellulose, which could hinder surface charge development. However, such a difference does not appear to significantly impact the practical stability of this suspension, particularly when compared to other samples under study.

In contrast, PE5, prepared under identical conditions (60 °C, 60 min, 50 mL H_2_SO_4_), displayed a zeta potential of −34.4 mV, demonstrating higher stability. The variability between these two samples may be attributed to inherent differences in precursor material composition or minor inconsistencies in local reaction environments. While this variation highlights the complexity of acid hydrolysis processes, both values are within the range expected for stable CNC dispersions, and the differences are not considered critical.

Under more intensive hydrolysis conditions, PE7, processed at 75 °C for 80 min with 75 mL of H_2_SO_4_, achieved a zeta potential of −33.4 mV. Although slightly lower than PE5, this value still reflects good colloidal stability. The higher temperature and acid concentration used in PE7 likely facilitated greater removal of amorphous regions and more extensive surface functionalisation, resulting in comparable stability to PE5 despite differing parameters [45,46]. This similarity between PE5 and PE7, despite their distinct conditions, suggests that certain stability thresholds can be reached under a variety of optimised parameter combinations.

The most stable samples, PE9 and PE18, exhibited zeta potentials of −37.4 mV and −37.3 mV, respectively. PE9 was processed at 75 °C for 40 min with 75 mL of H_2_SO_4_, while PE18 was processed at 81 °C for 60 min with 50 mL of H_2_SO_4_. These elevated zeta potential values indicate strong electrostatic repulsion, minimising aggregation and ensuring long-term dispersion stability. The higher temperature used in PE18 may have enhanced reaction kinetics, facilitating a more uniform removal of amorphous cellulose [47]. Meanwhile, the shorter reaction time for PE9, combined with a higher acid concentration, likely contributed to efficient surface functionalisation while avoiding excessive degradation of crystalline regions [48].

The differences in zeta potential across the samples reflect the interplay between reaction temperature, reaction time, and acid concentration. Higher temperatures, as seen in PE18 (81 °C, 60 min, 50 mL H_2_SO_4_), appear to promote greater surface charge density, likely due to more complete hydrolysis and enhanced exposure of functional groups. Similarly, higher acid quantities, as in PE9 (75 °C, 40 min, 75 mL H_2_SO_4_), may accelerate surface modification processes, achieving high zeta potential values even with shorter reaction times. The role of time is particularly evident when comparing these two samples. While PE18 demonstrates the benefits of prolonged hydrolysis at elevated temperatures, PE9 achieves comparable stability in a shorter time frame by utilising a higher acid concentration. This balance between temperature, acid concentration, and time is critical [49], as extended reaction times under intensive conditions could lead to the solubilisation or degradation of crystalline cellulose, negatively impacting colloidal stability.

Rice straw-derived cellulose, used as the starting material, already exhibits a high degree of purity with low contents of hemicellulose and lignin due to its prior processing [23]. However, it also contains significant amounts of amorphous cellulose [50,51], which requires careful optimisation of hydrolysis parameters for solubilisation. The elevated zeta potential values observed in PE9 (−37.4 mV) and PE18 (−37.3 mV) suggest how optimised hydrolysis conditions—whether through higher temperatures for longer durations or increased acid concentrations over shorter times—can effectively remove amorphous components while functionalising the CNC surfaces. These optimised conditions allow for the production of stable CNC suspensions with strong electrostatic repulsion, which are critical for ensuring long-term colloidal stability.

In summary, the zeta potential measurements reveal the significant role of hydrolysis conditions in determining CNC stability. PE9 and PE18, processed under distinct yet optimised conditions, emerged as the most stable samples, demonstrating the adaptability of acid hydrolysis to different processing goals. By fine-tuning these parameters, it is possible to achieve a balance that maximises stability and functional performance, making these CNCs suitable for applications requiring homogeneous and long-term dispersions.

**Figure 3:**
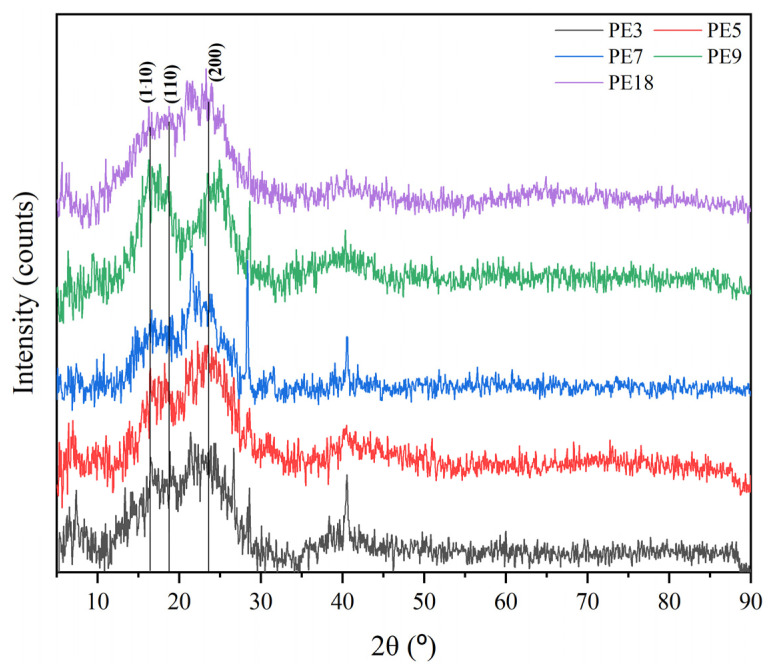
XRD patterns of cellulose nanocrystals obtained from rice straw under different acid hydrolysis conditions. The characteristic peaks of cellulose Iβ at ~18° and ~22° (2θ), corresponding to the (110) and (200) planes, respectively, are highlighted.

The colloidal stability findings were further contextualised by examining the crystalline properties of the CNCs through X-ray diffraction (XRD). This technique provided some insights into the structural order of the nanocrystals, particularly the crystallinity index (CI) and average crystallite size (CS). Table 3 summarises the results, highlighting the differences in crystalline structure among the samples and their correlation with the observed colloidal stability. Likewise, Figure 3 shows the diffractograms for each studied sample. The characteristic diffraction peaks of cellulose Iβ, located at approximately 2θ = 18° and 22°, corresponding to the (110) and (200) planes, respectively, are also indicated in Figure 3.

The XRD diffractogram of PE3, prepared under mild hydrolysis conditions (60 °C, 60 min, 50 mL H_2_SO_4_), revealed the lowest crystallinity index (50.2%) and the smallest crystallite size (1.2 nm). Although these results indicate a slightly lower degree of structural order compared to the other samples, the difference is minimal, suggesting that PE3 retains sufficient crystalline structure for stable dispersion. Its moderate zeta potential (−29.0 mV) reflects this balance, as it approaches the threshold for colloidal stability, indicating a performance aligned with its structural characteristics. The small differences in crystallinity and zeta potential between PE3 and PE5 could stem from inherent variability in the precursor material or slight inconsistencies in the reaction environment that affect amorphous cellulose removal and crystalline alignment. This highlights the sensitivity of both colloidal and crystalline properties to small changes in processing conditions.

On the other hand, PE5, processed under identical conditions to PE3 (60 °C, 60 min, 50 mL H_2_SO_4_), exhibited a slightly higher crystallinity index (51.0%) and a significantly larger crystallite size (1.7 nm). The slight improvement in structural parameters observed in PE5 compared to PE3 may reflect subtle differences in reaction kinetics at the microscale, which are challenging to control in larger-scale processes. These minor variations could have influenced the degree of hydrolysis and alignment of crystalline regions, leading to the improved crystallinity and zeta potential (−34.4 mV). While these differences are not dramatic, they underscore the need for precision in reaction conditions to achieve reproducibility in CNC properties.

PE7, processed under more intensive conditions (75 °C, 80 min, 75 mL H_2_SO_4_), demonstrated the highest crystallinity index (55.4%) and a relatively large crystallite size (1.7 nm). These results indicate a well-ordered crystalline structure, likely due to the higher temperature and acid concentration facilitating the efficient removal of amorphous regions and enhancing crystalline alignment. The zeta potential of PE7 (−33.4 mV) reflects good colloidal stability, though slightly lower than PE5. This suggests that the higher crystallinity in PE7 may have reduced the availability of surface functional groups responsible for charge generation, illustrating a trade-off between structural order and surface charge density [52].

PE9 and PE18, processed at 75 °C for 40 min with 75 mL H_2_SO_4_ and 81 °C for 60 min with 50 mL H_2_SO_4_, respectively, displayed balanced crystalline and colloidal properties. The crystallinity indices of PE9 (51.3%) and PE18 (51.6%) were slightly lower than that of PE7, but their zeta potentials (−37.4 mV and −37.3 mV, respectively) were the highest among all samples. These results suggest that the shorter reaction time of PE9 and the elevated temperature of PE18 promoted efficient surface functionalisation without compromising crystalline integrity. The smaller crystallite size of PE18 (1.3 nm) compared to PE9 (1.5 nm) may provide additional flexibility, making it suitable for applications requiring enhanced dispersion and adaptability [17,53]. Conversely, the larger crystallite size of PE9 could contribute to improved stiffness and structural reinforcement in composite materials [53].

A closer examination of the interplay between colloidal stability and crystallinity revealed a subtle but significant trade-off between these properties. For instance, while PE7 exhibited the highest crystallinity index, its zeta potential was slightly lower than that of PE9 and PE18. This could be attributed to the higher crystallinity reducing the number of available surface functional groups, thereby impacting charge density. Conversely, the slightly lower crystallinity indices of PE9 and PE18 were offset by their superior zeta potentials, indicating that their hydrolysis conditions promoted optimal surface modification while maintaining a reasonable degree of crystalline order.

These findings collectively underscore the critical role of hydrolysis conditions in determining the structural and colloidal properties of CNCs. Elevated temperatures and tailored reaction times, as demonstrated in PE9 and PE18, contribute to a balanced combination of high colloidal stability and adequate crystallinity [47,54]. In contrast, suboptimal conditions, such as those applied in PE3, may result in reduced structural order and stability, limiting the sample’s suitability for high-performance applications. These insights highlight the importance of optimising hydrolysis parameters to achieve specific property profiles tailored to intended applications.

**Figure 4:**
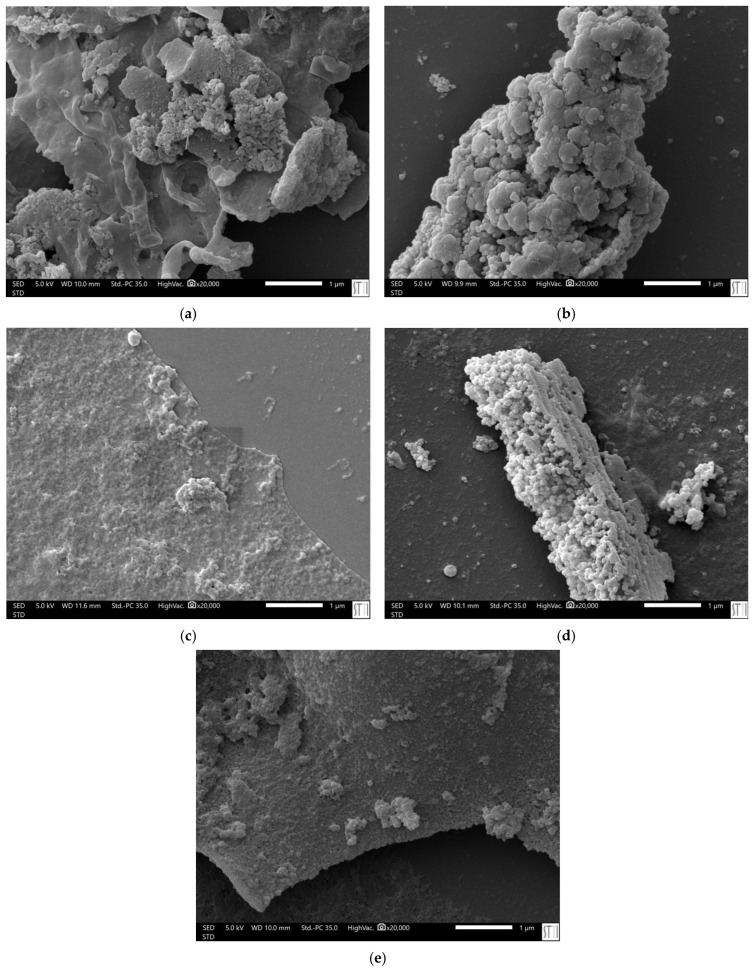
SEM images at ×20,000 magnification of CNCs obtained from rice straw cellulose under different acid hydrolysis conditions. Being (**a**) PE3, (**b**) PE5, (**c**) PE7, (**d**) PE9, and (**e**) PE18.

To further validate these results, the morphology of the cellulose nanocrystals (CNCs) obtained under varying acid hydrolysis conditions was assessed via SEM imaging, as shown in Figure 4. These micrographs reveal significant differences in the surface structure and aggregation of the CNCs, influenced by the hydrolysis parameters, particularly temperature, time, and acid concentration.

The SEM images of samples PE3 (60 °C, 60 min, 50 mL H_2_SO_4_) and PE5, prepared under identical conditions, reveal comparable morphological features, characterised by some aggregation and heterogeneous particle sizes. The minor differences observed between these two samples, such as slightly better-defined structures in PE5, align with their respective crystallinity indices (50.2% for PE3 and 51.0% for PE5) and zeta potential values (−29.0 mV and −34.4 mV). These differences are consistent with the inherent variability in the rice straw precursor or subtle localised effects during the reaction, which can marginally influence hydrolysis efficiency and the alignment of crystalline regions. Importantly, these findings suggest that the hydrolysis process under mild conditions yields CNCs with consistent and reproducible properties, as evidenced by the close proximity of their structural and colloidal characteristics.

PE7 (75 °C, 80 min, 75 mL H_2_SO_4_) displays nanocrystals with better structural definition compared to PE3 and PE5, though the particles remain compact. The higher crystallinity index (55.4%) and stable zeta potential (−33.4 mV) of PE7 suggest more effective removal of amorphous regions and improved surface repulsion, which contribute to the enhanced visibility of CNCs in this sample. PE9 (75 °C, 40 min, 75 mL H_2_SO_4_), however, emerges as the most distinct sample in SEM, with nanocrystals clearly visible and less compact than in PE7. This improved definition might be attributed to the balance between shorter reaction time and higher acid concentration, which could have facilitated optimal surface modification while preserving the structural integrity of CNCs [48].

Finally, PE18 (81 °C, 60 min, 50 mL H_2_SO_4_) exhibits compact structures similar to PE7, with observable CNCs that retain a degree of aggregation. The drying effects during SEM preparation might contribute to the clustering seen in PE18. However, the high zeta potential (−37.3 mV) and balance between hydrolysis conditions and surface charge indicate good dispersion properties in solution.

These results highlight the influence of hydrolysis conditions on both the structural and colloidal properties of CNCs. The SEM analysis supports the notion that higher temperatures and tailored acid concentrations promote the formation of more defined crystalline structures, although potential artefacts introduced during sample preparation must be considered when interpreting these findings.

**Figure 5:**
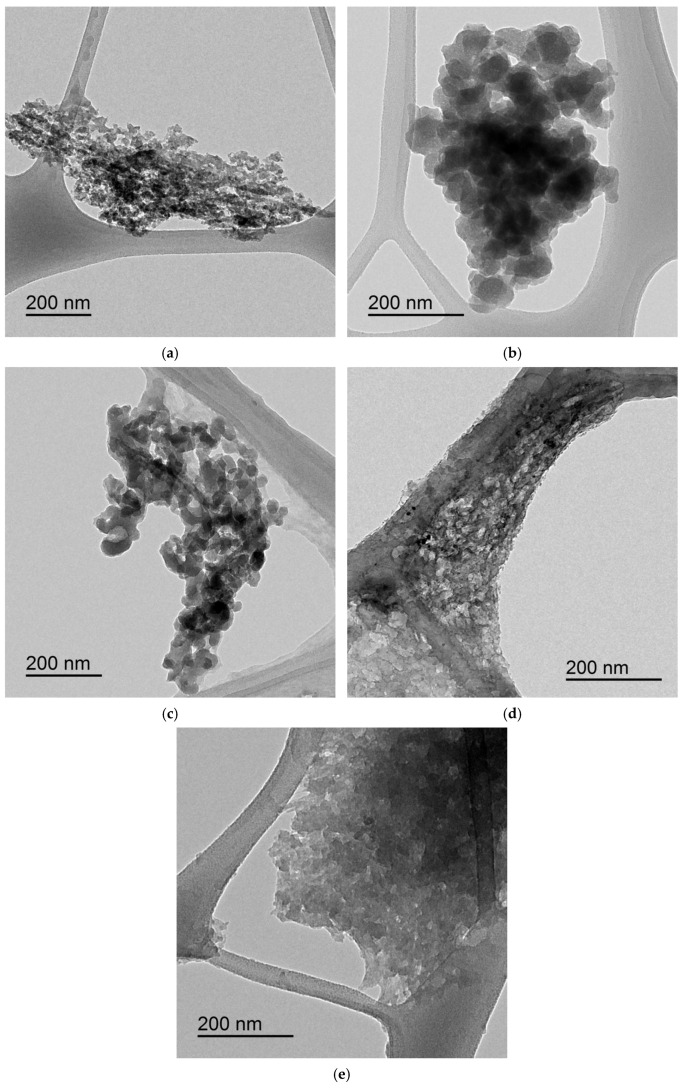
TEM images of CNCs obtained from rice straw cellulose under different acid hydrolysis conditions. Being (**a**) PE3, (**b**) PE5, (**c**) PE7, (**d**) PE9, and (**e**) PE18.

Further insight into the structural characteristics of the CNCs was obtained via TEM, as depicted in Figure 5. The high-resolution TEM images allow for a closer examination of individual nanocrystals, providing detailed information on their size, shape, and aggregation behaviour.

Samples treated under milder hydrolysis conditions, such as PE3 (60 °C, 60 min, 50 mL H_2_SO_4_), display a heterogeneous morphology, with poorly defined structures and variable particle sizes. This heterogeneity reflects the lower crystallinity index and zeta potential of PE3, which are indicative of incomplete hydrolysis and limited structural order. In contrast, PE5 (60 °C, 60 min, 50 mL H_2_SO_4_) shows clearer rod-like structures, confirming the formation of CNCs under these conditions. The dimensions of CNCs in PE5 are estimated to range between 30 and 100 nm, further validating the classification of these particles as nanocrystals.

PE7 (75 °C, 80 min, 75 mL H_2_SO_4_) similarly exhibits well-defined CNCs with minimal aggregation and consistent rod-like structures. Together with PE5, these samples demonstrate the most regular and clear CNC morphologies in TEM, reinforcing the role of processing parameters in achieving high structural order.

PE9 (75 °C, 40 min, 75 mL H_2_SO_4_), despite its clarity in SEM, shows more pronounced aggregation in TEM. This discrepancy might arise from preparation effects, such as drying-induced clustering, or from incomplete removal of amorphous regions during hydrolysis. The aggregated structures in PE9, while still indicative of nanocrystalline cellulose, appear less uniform compared to PE5 and PE7.

Finally, PE18 (81 °C, 60 min, 50 mL H_2_SO_4_) displays a mix of individual CNCs and aggregates, with rod-like structures visible within the clusters. The aggregation observed in PE18 may be partially attributed to sample preparation for TEM analysis, as drying effects can promote particle clustering. Nonetheless, the structural characteristics of PE18 are consistent with its high zeta potential and balanced hydrolysis conditions.

In summary, the results presented here provide a comprehensive understanding of the influence of acid hydrolysis conditions on the stability, crystallinity, and morphology of CNCs derived from rice straw. The correlation between zeta potential and XRD findings highlights the balance between colloidal stability and crystalline structure, while SEM and TEM analyses further confirm the role of hydrolysis parameters in defining CNC morphology. Milder conditions produce CNCs with moderate stability and heterogeneous structures, whereas more intensive treatments yield enhanced crystallinity, surface charge, and better-defined nanocrystals, though often accompanied by increased aggregation. These findings establish a robust framework for optimising CNC production, paving the way for tailored applications ranging from composite reinforcement to advanced coatings.

## 4. Conclusions

This study has thoroughly explored the effects of acid hydrolysis parameters on the production and properties of cellulose nanocrystals (CNCs) derived from rice straw. By systematically correlating zeta potential, structural observations from SEM and TEM, and crystallinity, a detailed understanding of the interplay between colloidal stability, structural order, and morphological characteristics has been achieved.

Milder hydrolysis conditions (60 °C, 60 min, 50 mL H_2_SO_4_) yielded CNCs with heterogeneous structures and moderate zeta potential (−29.0 mV). Increasing the temperature and acid concentration to 75 °C, 80 min, and 75 mL H_2_SO_4_ resulted in well-defined nanocrystals with improved colloidal stability (−33.4 mV). Optimised conditions such as 75 °C, 40 min, and 75 mL H_2_SO_4_ or 81 °C, 60 min, and 50 mL H_2_SO_4_ yielded CNCs with strong zeta potential (−37.4 mV and −37.3 mV, respectively), stable dispersions, and better-defined morphologies.

Starting from rice straw cellulose obtained through optimised pre-treatment methods, this study successfully demonstrates the transformation of this renewable resource into cellulose nanocrystals. These findings not only provide valuable insights into the optimisation of CNC production but also lay a foundation for their potential application in sectors such as footwear manufacturing, where stable dispersions and enhanced structural properties can contribute to reinforcing sustainable composites.

This work underscores the value of tailoring hydrolysis conditions to produce high-quality CNCs and aligns with circular economy principles, demonstrating how agricultural waste can be transformed into advanced, sustainable materials for industrial applications.

## Figures and Tables

**Table 1:** Tests carried out in a randomised manner based on the proposed statistical CCD design.

Sample	Temperature (°C)	Time (min)	H_2_SO_4_ 50% (mL)
PE1	39	60	50.0
PE2	60	60	85.4
PE3	60	60	50.0
PE4	75	80	25.0
PE5	60	60	50.0
PE6	45	40	25.0
PE7	75	80	75.0
PE8	45	80	75.0
PE9	75	40	75.0
PE10	60	60	50.0
PE11	45	80	25.0
PE12	75	40	25.0
PE13	60	60	14.7
PE14	60	60	50.0
PE15	60	88	50.0
PE16	60	32	50.0
PE17	45	40	75.0
PE18	81	60	50.0

**Table 2:** Zeta potential values of the selected CNC samples.

Sample	Z Potential (mV)
PE3	−29.0
PE5	−34.4
PE7	−33.4
PE9	−37.4
PE18	−37.3

**Table 3:** Crystallinity index (CI) and crystallite size (CS) of the selected CNC samples.

Sample	Crystallinity Index (%)	Crystallite Size (nm)
PE3	50.2	1.2
PE5	51.0	1.7
PE7	55.4	1.7
PE9	51.3	1.5
PE18	51.6	1.3

## Data Availability

The original contributions presented in the study are included in the article/Supplementary Material, further inquiries can be directed to the corresponding authors.

## Supplementary Materials

The following supporting information can be downloaded at: https://www.mdpi.com/article/10.3390/polym17101313/s1.

## References

[1] Sharma P.R., Huang X., Yang M., Sharma S.K., Hsiao B.S. (2022). Cellulose Nanofibers for Sustainable Separations. Sustainable Separation Engineering.

[2] Ghaffar A., Salman M., Yameen M., Iqbal S.Z., Altaf S., Munir B. (2023). Sustainable Biomedical Applications of Cellulose. Regenerated Cellulose and Composites.

[3] Kramar A., González-Benito F.J. (2022). Cellulose-Based Nanofibers Processing Techniques and Methods Based on Bottom-Up Approach—A Review. Polymers.

[4] Habibi Y., Lucia L.A., Rojas O.J. (2010). Cellulose Nanocrystals: Chemistry, Self-Assembly, and Applications. Chem. Rev..

[5] Phuong H.T., Thoa N.K., Tuyet P.T.A., Van Q.N., Hai Y.D. (2022). Cellulose Nanomaterials as a Future, Sustainable and Renewable Material. Crystals.

[6] Carolin C.F., Kamalesh T., Kumar P.S., Hemavathy R.V., Rangasamy G. (2023). A Critical Review on Sustainable Cellulose Materials and Its Multifaceted Applications. Ind. Crops Prod..

[7] Wasim M., Shi F., Liu J., Khan M.R., Farooq A., Sanbhal N., Alfred M., Xin L., Yajun C., Zhao X. (2021). Extraction of Cellulose to Progress in Cellulosic Nanocomposites for Their Potential Applications in Supercapacitors and Energy Storage Devices. J. Mater. Sci..

[8] Trache D., Tarchoun A.F., Abdelaziz A., Bessa W., Hussin M.H., Brosse N., Thakur V.K. (2022). Cellulose Nanofibrils–Graphene Hybrids: Recent Advances in Fabrication, Properties, and Applications. Nanoscale.

[9] Jenol M.A., Norrrahim M.N.F., Nurazzi N.M. (2022). Nanocellulose Nanocomposites in Textiles. Industrial Applications of Nanocellulose and Its Nanocomposites.

[10] Gabriel T., Belete A., Hause G., Neubert R.H.H., Gebre-Mariam T. (2021). Isolation and Characterization of Cellulose Nanocrystals from Different Lignocellulosic Residues: A Comparative Study. J. Polym. Environ..

[11] Ma L., Xu Y., Chen J., Dong C., Pang Z. (2023). Preparation of Cellulose Nanocrystals by Synergistic Action of Ionic Liquid and Recyclable Solid Acid under Mild Conditions. Molecules.

[12] Rovera C., Carullo D., Bellesia T., Büyüktaş D., Ghaani M., Caneva E., Farris S. (2023). Extraction of High-Quality Grade Cellulose and Cellulose Nanocrystals from Different Lignocellulosic Agri-Food Wastes. Front. Sustain. Food Syst..

[13] Anusiya G., Jaiganesh R. (2022). A Review on Fabrication Methods of Nanofibers and a Special Focus on Application of Cellulose Nanofibers. Carbohydr. Polym. Technol. Appl..

[14] Nasution H., Yahya E.B., Abdul Khalil H.P.S., Shaah M.A., Suriani A.B., Mohamed A., Alfatah T., Abdullah C.K. (2022). Extraction and Isolation of Cellulose Nanofibers from Carpet Wastes Using Supercritical Carbon Dioxide Approach. Polymers.

[15] Kerwald J., de Moura Junior C.F., Freitas E.D., de Moraes Segundo J.d.D.P., Vieira R.S., Beppu M.M. (2022). Cellulose-Based Electrospun Nanofibers: A Review. Cellulose.

[16] Djafari Petroudy S.R., Chabot B., Loranger E., Naebe M., Shojaeiarani J., Gharehkhani S., Ahvazi B., Hu J., Thomas S. (2021). Recent Advances in Cellulose Nanofibers Preparation through Energy-Efficient Approaches: A Review. Energies.

[17] Mohomane S.M., Motloung S.V., Koao L.F., Motaung T.E. (2022). Effects of acid hydrolysis on the extraction of cellulose nanocrystals (cncs): A review. Cellul. Chem. Technol..

[18] Samarawickrama K.G.R., Wijayapala U.G.S., Fernando C.A.N. (2023). Extraction and Analysis of Cellulose Nanocrystals from Cotton Balls by Acid Hydrolysis. J. Sci. Univ. Kelaniya.

[19] Hernández Pérez R., Salgado Delgado R., Olarte Paredes A., Salgado Delgado A., García Hernández E., Medrano Valis A., Martínez Candia F. (2022). Comparing Acid and Enzymatic Hydrolysis Methods for Cellulose Nanocrystals (CNCs) Obtention from Agroindustrial Rice Husk Waste. J. Nanotechnol..

[20] Xie H., Du H., Yang X., Si C. (2018). Recent Strategies in Preparation of Cellulose Nanocrystals and Cellulose Nanofibrils Derived from Raw Cellulose Materials. Int. J. Polym. Sci..

[21] Jordan J.H., Easson M.W., Condon B.D. (2020). Cellulose Hydrolysis Using Ionic Liquids and Inorganic Acids under Dilute Conditions: Morphological Comparison of Nanocellulose. RSC Adv..

[22] Pantamanatsopa P., Ariyawiriyanan W., Ekgasit S. (2023). Production of Cellulose Nanocrystals Suspension with High Yields from Water Hyacinth. J. Nat. Fibers.

[23] Serrano-Martínez V.M., Pérez-Aguilar H., Carbonell-Blasco M.P., Arán-Ais F., Orgilés-Calpena E. (2024). Steam Explosion-Based Method for the Extraction of Cellulose and Lignin from Rice Straw Waste. Appl. Sci..

[24] Moon R.J., Martini A., Nairn J., Simonsen J., Youngblood J. (2011). Cellulose Nanomaterials Review: Structure, Properties and Nanocomposites. Chem. Soc. Rev..

[25] Douglas C. (2012). Montgomery Response Surface Methods and Designs. Design and Analysis of Experiments.

[26] Purwanti E., Dampang S. (2017). Pengaruh Perbedaan Kondisi Hidrolisis Terhadap Hasil Isolasi Nanokristalin Selulosa Dari Bonggol Jagung. Indo. J. Chem. Res..

[27] Johar N., Ahmad I., Dufresne A. (2012). Extraction, Preparation and Characterization of Cellulose Fibres and Nanocrystals from Rice Husk. Ind. Crops Prod..

[28] Putri E., Gea S. (2018). Isolasi Dan Karakterisasi Nanokistral Selulosa Dari Tandan Sawit (Elaeis Guineensis Jack). Elkawnie.

[29] Phanthong P., Reubroycharoen P., Hao X., Xu G., Abudula A., Guan G. (2018). Nanocellulose: Extraction and Application. Carbon Resour. Convers..

[30] Wickaramasinghe W.A.W.I.C., Lasitha D.S., Samarasekara A.M.P.B., Amarasinghe D.A.S., Karunanayake L. Extraction and Characterization of Nano Crystalline Cellulose (NCC) From Sri Lankan Agricultural Waste. Proceedings of the 2019 Moratuwa Engineering Research Conference (MERCon).

[31] Henrique M.A., Flauzino Neto W.P., Silvério H.A., Martins D.F., Gurgel L.V.A., Barud H.d.S., de Morais L.C., Pasquini D. (2015). Kinetic Study of the Thermal Decomposition of Cellulose Nanocrystals with Different Polymorphs, Cellulose I and II, Extracted from Different Sources and Using Different Types of Acids. Ind. Crops Prod..

[32] Dias Y.J., Silva V.D., Pourdeyhimi B., Medeiros E.S., Yarin A.L. (2023). Freestanding Carbon Nanofibers Derived from Biopolymer (Kraft Lignin) as Ultra-Microporous Electrodes for Supercapacitors. Batteries.

[33] Ashikbayeva Z., Aitkulov A., Jelbuldina M., Issatayeva A., Beisenova A., Molardi C., Saccomandi P., Blanc W., Inglezakis V.J., Tosi D. (2020). Distributed 2D Temperature Sensing during Nanoparticles Assisted Laser Ablation by Means of High-Scattering Fiber Sensors. Sci. Rep..

[34] Carbonell-Blasco P., Martín-Martínez J.M., Antoniac I.V. (2013). Synthesis and Characterization of Polyurethane Sealants Containing Rosin Intended for Sealing Defect in Annulus for Disc Regeneration. Int. J. Adhes. Adhes..

[35] Khan A., Toufiq A.M., Tariq F., Khan Y., Hussain R., Akhtar N., Rahman S. (2019). ur Influence of Fe Doping on the Structural, Optical and Thermal Properties of *α*-MnO_2_ Nanowires. Mater. Res. Express.

[36] Vinila V.S., Isac J. (2022). Synthesis and Structural Studies of Superconducting Perovskite GdBa2Ca3Cu4O10.5+δ Nanosystems. Design, Fabrication, and Characterization of Multifunctional Nanomaterials.

[37] Abou-Sekkina M.M., Sakran M.A., Saafan A.A. (1986). Development of Correlations among the Spectral, Structural, and Electrical Properties and Chemical Treatment of Egyptian Cotton Fabric Strips. Ind. Eng. Chem. Prod. Res. Dev..

[38] French A.D. (2014). Idealized Powder Diffraction Patterns for Cellulose Polymorphs. Cellulose.

[39] (2015). Statgraphics Technologies STATGRAPHICS^®^ Centurion XVII Manual de Usuario. https://www.statgraphics.net/wp-content/uploads/2015/03/Centurion-XVII-Manual-Principal.pdf.

[40] Douglas C. (2019). Montgomery. Design and Analysis of Experiments.

[41] Batanero C., Díaz Batanero M.C. (2008). Análisis de Datos Con Statgraphics.

[42] Leenen I. (2012). La Prueba de La Hipótesis Nula y Sus Alternativas: Revisión de Algunas Críticas y Su Relevancia Para Las Ciencias Médicas. Investig. En Educ. Medica.

[43] Feng Y., Kilker S.R., Lee Y. (2020). Surface Charge (Zeta-Potential) of Nanoencapsulated Food Ingredients. Characterization of Nanoencapsulated Food Ingredients.

[44] Németh Z., Csóka I., Semnani Jazani R., Sipos B., Haspel H., Kozma G., Kónya Z., Dobó D.G. (2022). Quality by Design-Driven Zeta Potential Optimisation Study of Liposomes with Charge Imparting Membrane Additives. Pharmaceutics.

[45] Alhaji Mohammed M., Basirun W.J., Abd Rahman N.M.M., Shalauddin M., Salleh N.M. (2022). The Effect of Acid Hydrolysis Parameters on the Properties of Nanocellulose Extracted from Almond Shells. J. Nat. Fibers.

[46] Ioelovich M. (2014). Peculiarities of Cellulose Nanoparticles. Tappi J..

[47] Kanchanalai P., Temani G., Kawajiri Y., Realff M.J. (2016). Reaction Kinetics of Concentrated-Acid Hydrolysis for Cellulose and Hemicellulose and Effect of Crystallinity. Bioresources.

[48] Jannah A.N., Fuadi A.M. (2022). Effect of Hydrolysis Time and Sulfuric Acid Concentration on Reducing Sugar Content on Corn Cob Hydrolysis. Chem. J. Tek. Kim..

[49] Rana M.S., Rahim M.A., Mosharraf M.P., Tipu M.F.K., Chowdhury J.A., Haque M.R., Kabir S., Amran M.S., Chowdhury A.A. (2023). Morphological, Spectroscopic and Thermal Analysis of Cellulose Nanocrystals Extracted from Waste Jute Fiber by Acid Hydrolysis. Polymers.

[50] Zhao G., Du J., Chen W., Pan M., Chen D. (2019). Preparation and Thermostability of Cellulose Nanocrystals and Nanofibrils from Two Sources of Biomass: Rice Straw and Poplar Wood. Cellulose.

[51] Sharma N., Allardyce B.J., Rajkhowa R., Agrawal R. (2023). Rice Straw-Derived Cellulose: A Comparative Study of Various Pre-Treatment Technologies and Its Conversion to Nanofibres. Sci. Rep..

[52] Whba F., Mohamed F., Idris M.I., Yahya M.S. (2022). Characterization of Cellulose Nanocrystals (CNCs) Derived from Microcrystalline Cellulose (MCC) Synthesized Using Acid Hydrolysis Method. https://www.researchsquare.com/article/rs-2078344/v2.

[53] Sarı B., Kaynak C. (2024). Obtaining Cellulose Nanocrystals by Acid Hydrolysis Procedure; and Their Use as Reinforcement in Polylactide Biocomposites. J. Thermoplast. Compos. Mater..

[54] Kargarzadeh H., Ioelovich M., Ahmad I., Thomas S., Dufresne A. (2017). Methods for Extraction of Nanocellulose from Various Sources. Handbook of Nanocellulose and Cellulose Nanocomposites.

