# Supplementary material for: Effect of Acid Hydrolysis Conditions on the Extraction of Cellulose Nanocrystals
Source: Polymers (Basel). 2025 May 12;17(10):1313. doi: 10.3390/polym17101313 (PMC12115138; doi:10.3390/polym17101313)
Supplement: Supplementary file 1 [file polymers-17-01313-s001.zip › polymers-3635182-supplementary.pdf]

Supplementary material.

Effect of Acid Hydrolysis Conditions on the Extraction of Cellulose Nanocrystals

In an attempt to obtain information about the crystalline structure of the dispersed samples, XRD analyses were conducted as an approach for the obtention of parameters reported in literature from which is plausible to discern crystalline features of these solids. For this purpose, the suspensions were dried just before the XRD measurements. As expected, it can be inferred a low degree of crystallinity for all the suspensions analysed (from the low ratio signal-noise obtained and clearly seen in the representations), but, still, some authors report the estimations of parameters to provide relative differences among the samples obtained. It is worth noting the majority presence of β phase (whose main reflections are depicted in the ICDD file 00-050-2241, with peaks (1-10) at 14.8°, (110) at 16.7 and (200) at 22.8°, as shown in Figure 3 in the article). Nevertheless, the resolution of the main reflections in distinct peaks does not take place due to the very low degree of crystallinity of the samples.

For this purpose and taking into account the interest of comparing structural data with other properties herein presented, the crystallinity index (CI) was calculated using the methodology described by Khan et al. [reference 33 in the article]. This index was determined by extracting individual crystalline peaks through a curve-fitting process from diffraction intensity profiles, applying Gaussian functions for each peak until achieving a high correlation coefficient (R^2 value of 0.997). The average crystallite size was calculated using the Scherrer equation [reference 34 in the article], which is widely recognised for estimating crystallite sizes in nanocrystalline samples. The indexed diffractograms correspond to the cellulose Iβ phase (ICDD file 00-050-2241) [reference 36 in the article].
